# Time-to-Treatment in Oral Cancer: Causes and Implications for Survival

**DOI:** 10.3390/cancers13061321

**Published:** 2021-03-16

**Authors:** Constanza Saka-Herrán, Enric Jané-Salas, Antoni Mari-Roig, Albert Estrugo-Devesa, José López-López

**Affiliations:** 1 Department of Odontostomatology, Faculty of Medicine and Health Sciences (Dentistry), University of Barcelona, 08970 Barcelona, Spain; csakaher20@alumnes.ub.edu; 2 Oral Health and Masticatory System Group (Bellvitge Biomedical Research Institute) IDIBELL, Faculty of Medicine and Health Sciences (Dentistry), University of Barcelona, 08970 Barcelona, Spain; enricjanesalas@ub.edu (E.J.-S.); amari@bellvitgehospital.cat (A.M.-R.) albertestrugo@ub.edu (A.E.-D.); 3 Service of the Medical-Surgical Area of Dentistry Hospital, University of Barcelona, 08970 Barcelona, Spain

**Keywords:** head and neck cancer, oral cancer, time-to-treatment, treatment delays, early diagnosis, survival rate

## Abstract

**Simple Summary:**

Stage of the disease at diagnosis has been recognized as one of the most important prognostic markers for oral cancer. Unfortunately, still two thirds of patients are diagnosed at an advanced stage of disease with a 5-year survival rate of 50% or less. Although the detection of oral cancer at an early stage is the most effective means to improve survival and reduce morbidity, in the past years, there has been little change in the diagnosis of oral cancer at early stages, which is believed to be a result of delays in diagnosis and treatment of oral cancer, among other independent factors. Following the Aarhus statement, developed in effort to standardize the design, methods and reporting of studies concerning time-intervals in early diagnosis research, the review assessed the causes that influence the patient, diagnosis and pre-treatment intervals in the pathway of time-to-treatment in oral cancer and its impact on survival.

**Abstract:**

The purpose of this review was to identify and describe the causes that influence the time-intervals in the pathway of diagnosis and treatment of oral cancer and to assess its impact on prognosis and survival. The review was structured according to the recommendations of the Aarhus statement, considering original data from individual studies and systematic reviews that reported outcomes related to the patient, diagnostic and pre-treatment intervals. The patient interval is the major contributor to the total time-interval. Unawareness of signs and/or symptoms, denial and lack of knowledge about oral cancer are the major contributors to the process of seeking medical attention. The diagnostic interval is influenced by tumor factors, delays in referral due to higher number of consultations and previous treatment with different medicines or dental procedures and by professional factors such as experience and lack of knowledge related to the disease and diagnostic procedures. Patients with advanced stage disease, primary treatment with radiotherapy, treatment at an academic facility and transitions in care are associated with prolonged pre-treatment intervals. An emerging body of evidence supports the impact of prolonged pre-treatment and treatment intervals with poorer survival from oral cancer.

## 1. Introduction

In 2018 oral cancer accounted for 354,864 (2%) new cases and 177,384 (1.9%) deaths worldwide, with the highest incidence in Southern Asia and the Pacific Islands and being the eighth most common type of cancer in men [1]. Despite advances in diagnosis and oncologic treatment during the last decades, the 5-year survival rates of oral cancer still remain in the 50–60% range [2,3], with a slight increase observed in the United States (US) during the last decade (66%) [4].

Several factors have been assessed as independent prognostic factors for head and neck cancer (HNC). These factors include demographic and patient factors, lifestyle factors, treatment modality factors and tumor factors [5]. However, of the prognostic factors, tumor size and increased stage, nodal involvement (extracapsular spread), distant metastasis, positive margins and, consequently, the stage of the presenting lesion at diagnosis are the most important prognostic markers for oral cancer [2,5,6]. Unfortunately, still two thirds of patients with oral cancer are diagnosed at an advanced stage of disease (stage III and IV) [3,7] with a 5-year survival rate of 50% or less [2] compared to the more than 80% survival rate in those with localized disease, which make the differences in mortality rates based on staging very marked [8].

The detection of oral cancer at an early stage is the most effective means to improve survival and, at the same time, reduce morbidity, disfigurement, duration of treatment and hospital costs. It may also enhance psychological outcomes and quality of life [9,10]. However, according to the National Cancer Institute’s SEER program, in the past 10 years, only 28.7% of oral cavity and pharynx cancers were diagnosed at a local stage (stage I) [4], which is believed to be a result of the silent nature of early oral lesions and delay in diagnosis [11].

To date, there are no standardized definitions for describing delays in diagnosis and/or treatment of oral cancer, with diverse definitions existing among the literature to describe the patient’s pathway from their first awareness of symptoms to the initiation of treatment [12]. Moreover, the absence of a theoretical framework and the lack of a consensus on a time-point beyond which a cancer diagnosis should be considered as delayed [13] has led to the use of a wide range of endpoints for defining “delay” in research which is mostly based in arbitrary cut-off points.

**Figure 1:**
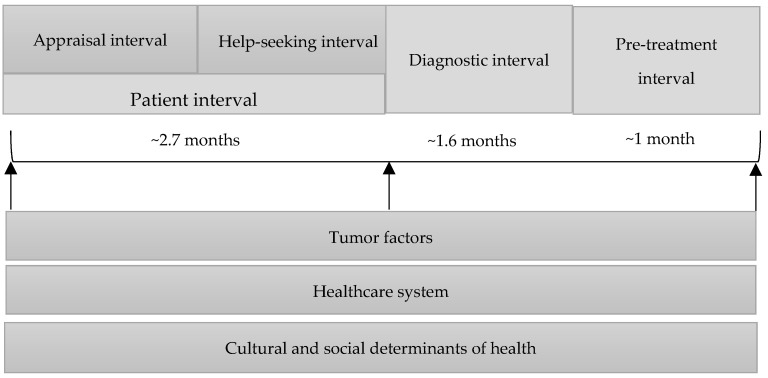
Time-intervals in the pathway of diagnosis and treatment of oral cancer.

In effort to standardize the design, methods and reporting of studies concerning time points and intervals in early-diagnosis research, the Aarhus statement has been proposed [14]. This guideline is a refined version derived from the Andersen Model of Total Patient Delay [15] which also recommends the replacement of the term “delay” for “time-intervals” as the former term is considered value laden and inaccurate [15]. The statement suggests the use of four key time points (date of first symptom, date of first presentation, date of referral and date of diagnosis) and their associated time-intervals, appraisal, help-seeking, diagnostic and pre-treatment intervals [14] to describe the patients pathway from first symptom awareness to the initiation of treatment, considering that all the process take place within a health system that can influence the length of time-intervals and so will have effects on all stages of this pathway [16]. Cultural and social determinants of health must also be taken into account as they also have an impact on all aspects of the length-time in treatment of oral cancer (Figure 1).

It has been reported that the mean total-interval, defined as the time between the patient first awareness of signs and/or symptoms until the initiation of treatment, is 206 days with a range of 52–786 days (~6.9 months) [17]. Consistently, a recent systematic review reported a mean total-interval of 186.7 days (~6.2 months), derived from data of four studies reporting on 527 patients [18].

Following the Aarhus statement, the purpose of this review is to identify and describe the causes that influence the length-time in treatment of oral cancer and assess its impact on prognosis and survival. For practical considerations, the appraisal and help-seeking intervals were reported in one interval, the patient interval, as many studies report outcomes for the time-interval between the patient first awareness of signs and/or symptoms and his first consultation to a healthcare provider.

## 2. Patient Interval

The patient interval is defined as the time between the patient first awareness of signs and/or symptoms and his first consultation to a healthcare provider (HCP) and comprises both the appraisal and help-seeking intervals [14,18]. It is reported to be the most significant contributor to the total time-interval [2,12,17], comprising 1.6 to 5.6 months approximately [7,12,19,20]. Peacock et al. [17] reported a mean patient interval of 3.5 months (104.7 days) in the US and similar results were described in a study conducted in German patients with HNC, where the time elapsed between the first symptoms and medical consultation for the majority of patients (63.5%) was 3–4 months [21]. On the other hand, results from a study conducted in Chinese patients with oral cancer reported a median patient interval of 1 month (30 days) [22] and similar results were reported in Iranian patients with a median interval of 1.5 months (45 days) [23]. The results from the systematic review published by Varela-Centelles et al. [18] described a weighted mean of 80.3 days (~2.7 months) calculated from 2151 patients, with the shortest intervals (28–42 days) reported from Europe (The Netherlands, Finland).

### Causes Influencing the Patient Interval

Studies evaluating sociodemographic variables such as age, gender, area of residence or religion have consistently shown a non-association with the patient interval [10,11,24]. It has also been reported that the patient interval is independent of health-related behaviors, i.e., tobacco and alcohol consumption [10,11,19] and of clinical/tumor factors [9,11]. Rather, the patient interval seems to be mainly related to cognitive and psychosocial variables [24]. Unawareness of signs and/or symptoms, nonpersonal experience of cancer, lack of knowledge about cancer and denial have been assessed as strongly independent factors associated to the patient interval [24].

Research on other diseases indicates that symptom appraisal is the most important stage in the process of seeking medical attention, constituting approximately 60% of the total route [9], being the non-recognition or misinterpretation of symptoms, mainly to the lack of knowledge [25], the most predominant risk factor [24]. A qualitative study reported that patients often attributed their symptoms to transient, minor conditions (i.e., mouth ulcer, physical trauma, dental problems) and, in turn, were unconcerned about their presence [26]. Hence, patient’s cognitive and emotional perceptions are determining factors in their decision to seek or not to seek help. The least common initial interpretation was cancer which may reflect the general lack of knowledge of the disease that may lead to misguided appraisal of their symptoms and thus can lead to inappropriate behavioral responses which may adversely affect help-seeking behavior [9,26]. In that context, Scott et al. [11] showed that knowledge of oral cancer in the UK was independently associated with the patient interval, indicating that participants who had more accurate knowledge of oral cancer were less likely to delay seeking help (OR: 0.75, 95%CI 0.57–0.98). Similarly, Zhang et al. [22] reported that patients in China with higher oral cancer knowledge had a lower possibility of delay in seeking professional advice, although not statistically significant (OR: 0.88, 95%CI 0.60–2.01). Reda et al. [27,28] demonstrated that a low health literacy was associated with a lower utilization of dental services, an indicator of healthcare access, (OR: 0.41, 95%CI 0.01–0.81) [28] and that participants with lower educational level had a significantly lower access to dental services compared to those with higher education level (OR: 0.61, 95%CI 0.55–0.68) [27]. Health literacy is closely related with oral health beneficial behavior and is associated with general education [28], thus conditioning the decision to seek medical attention which could also be influenced by educational barriers.

Moreover, there are large differences in access and utilization of dental services according to different social, demographic, economical or educational factors that can influence the prevention, diagnosis and management of oral diseases. It has been identified that access to dental services is lower in ethnic minorities or immigrants, in those living in rural places, those with lower educational and income level and among those without insurance coverage which reflects inequalities in access to dental care [27]. Scott et al. [29] also recognized problems with access to an HCP in the UK, which included the distance needed to travel and the cost of a consultation which were perceived as barriers for seeking medical attention. This population should also be targeted as a high-risk group, as their social, structural, demographical and economical barriers can decisively condition their timing of oral cancer diagnosis [30].

## 3. Diagnostic Interval

The diagnostic interval is defined as the time from first consultation by an HCP to achieving the definitive diagnosis [14,18] and it is where patients with asymptomatic lesions that are potentially able to be detected through opportunistic screening would enter the model’s pathway [31].

Length-time of diagnostic interval vary substantially across studies. Friedrich et al. [21] reported a mean diagnostic interval of 7.1 months in Germany, while Yu et al. [20] reported a median time-interval of 3 months in Canada, finding that initial treatment by general practitioners (GP) before referring to a specialist was associated with a higher diagnostic interval. In patients with newly diagnosed HNC, a median diagnostic interval of 14 days was reported by two studies in Europe [32,33], although one of them did not consider the time from first consultation by an HCP to the first visit to a specialized professional [32]. The pooled estimate of the aforementioned systematic review [18], comprising a total of 1324 patients with symptomatic oral lesions, reported a diagnostic interval of 48 days (~1.6 months). The authors identified that the longest intervals were reported in the US in the 1980s and more recently in Australia and Iran. On the other hand, the shortest diagnostic intervals (21–22 days) were reported from European countries [18].

### Causes Influencing the Diagnostic Interval

It has been reported that diagnostic interval is related with tumor factors such as stage at presentation and localization of the tumor with small tumors (T1–T2) and laryngeal tumors associated with a higher time-interval in diagnosis [32]. An explanation could be that diagnosis is easier when tumors are larger because they are visible and because the oral cavity is easily accessible for examination [32]. Other factors associated are a higher number of consultations (≥3) before achieving definitive diagnosis, previous treatment with different medicines (analgesics, antibiotics) and previous dental procedures [23]. The results of a previous systematic review reported that dentists were more likely to delay referral when compared to GP as they can first undertake dental procedures; still there was no clear difference in stage of the disease at diagnosis or delay in referral between the professionals [8].

Other causes related are low index of suspicion, lack of familiarity and experience with the disease and lack of oral cancer knowledge on diagnostic procedures, main locations of oral cancer and leuko- or erythroplakia-like carcinomas as primary oral cancer lesions [30]. Early diagnosis of oral pre-cancerous lesions is particularly challenging because it requires practitioners to be familiar with the range of clinical presentations of potentially malignant disorders, many of which may resemble less serious conditions [31]. In addition, dental and GP may not easily discriminate malignant lesions due to the low incidence of oral malignancies among the general population and the nonspecific appearance of the lesions, especially in young and low risk patients [7]. A recent systematic review stated that there was a low level of awareness among medical practitioners regarding common potential malignant oral lesions involving leukoplakia (56%), erythroplakia (30%) and oral lichen planus (13%) and a moderate knowledge about frequent sites of oral cancer development involving the tongue (48%) and floor of the mouth (37%). Moreover, only 27% of medical practitioners performed an intraoral examination as a routine [34]. This point to the key importance of recognition of abnormality; as without this, no further action would be taken [7].

Additionally, definitive diagnosis depends on diagnostic procedures such as detection of tissue change, decision to biopsy, biopsy site selection, quality of the tissue submitted, laboratory procedure and pathologist’s skill and interpretation. Consequently, each step-in patient presentation and professional decision making can contribute to increase the diagnostic time-interval [7]. Regarding the decision to biopsy, results from a study conducted in 121 Brazilian dentists showed that only 10.7% of the professionals routinely would perform biopsies and only 3.3% would perform in any situation (independent on the complexity) [35]. Anandani et al. [36] reported that 50.8% of dentists in India preferred to refer the patients to a specialist to perform the biopsy being the reasons for their reluctance the lack of instruments and experience required for taking biopsies. Also, 46.3% of the dentists sent the tissue for analysis only when suspecting premalignancy [36]. Results from a Spanish study indicated that 50% of dentists performed at least one biopsy per year to confirm or rule out oral cancer. Furthermore, the diagnostic sensitivity for oral cancer was 61.4% and 57.8% for the combined oral cancer and premalignant oral lesions [37]. Therefore, poor knowledge and confidence of practitioners in screening, biopsy and referral of suspicious lesions appear to be significant, but modifiable, negative influences in the early detection and diagnosis of oral cancer [31].

## 4. Pre-Treatment Interval

The pre-treatment interval is defined as the time from the diagnosis to the start of treatment [14] and may be influenced by the patient, the health system and lesion factors [15,31]. Results from a study conducted in the United States (US), that used the National Cancer Data Base, reported a median pre-treatment interval of 30 days [38]. Similarly, Kaing et al. [39] reported a median time from diagnosis to definitive treatment of 30 days (range 0–9 weeks) in 101 Australian patients firstly diagnosed with OSCC and Lyhne et al. [33] a median time-interval of 25 days in Denmark. The results provided showed a significant reduction in pre-treatment waiting time for HNC patients of 41% from 2002 to 2010, corresponding to a reduction of four weeks with the most pronounced reduction seen in waiting time for definitive radiotherapy that decreased 3 weeks [33]. The authors concluded that the implementation of a fast-track policy in Denmark in 2007 could be an important contributing factor for the decrease [33]. Contrary, in the US an increase of 58% in the pre-treatment interval was found between 1998 (19 days) and 2011 (30 days) with an overall median of 26 days [40]. The authors discussed that the trend in rising could possibly be due to increases in sophistication and number of pre-treatment radiologic/pathologic testing, increases in the use and complexity of multimodality therapies and increased transitions in care [40].

### Causes Influencing the Pre-Treatment Interval

Patient factors associated with an increased pre-treatment interval are age ≥60 years and concurrent comorbidity [38,41]. The presence of comorbidities may require optimization before initiation of surgical or medical oncologic therapy which may lengthen the diagnosis-to-treatment time [41]. Treatment at an academic/research facility (educational institutions) and diagnosis at an outside facility has also been associated with an increase pre-treatment interval [38]. In that context, Murphy et al. [40] reported that treatment at an academic facility in the US was associated with a higher time-interval in treatment initiation (median = 35 days) compared to treatment at a community facility (median = 28 days) and transitions in care, defined as a change in facility from diagnosis to definitive treatment, was also associated with a significantly longer median pre-treatment interval. Similarly, van Harten et al. [42] described a longer pre-treatment interval in patients in The Netherlands who experienced transitions in care (median = 44 days) compared to those diagnosed and treated in the same center (median = 31 days), probably attributed to a delay in referral. Other factors significantly associated with a prolonged pre-treatment interval were African American race, Hispanic ethnicity, lack of insurance or Medicaid coverage, lower education levels and distance of primary residence from treatment facility [40,43], all of which reflect the barriers and difficulties of social determinants of health in the initial access to healthcare.

Patients with advanced stage disease has also been significantly associated with a longer pre-treatment interval [40,41,42]. Significant differences have also been reported between treatment modalities, were patients treated with primary radiotherapy or chemoradiation experienced a longer diagnosis-to-treatment interval (median = 57 days [41], median = 42 days [42]) compared to patients surgically treated (median = 30 days [41], median = 30 days [42]). Patients at an advanced stage disease require a multidisciplinary treatment approach with the involvement of multiple subspeciality physicians and oncologic specialists before initiation of treatment as well as the frequent need of dental visits for assessment of oral health status and dental extractions for improvement of their oral health condition before treatment or radiotherapy initiation, therefore, lengthening the time-to-treatment initiation [41].

Finally, institution-based factors affecting timely delivery of oncologic care include availability of specialty service, outpatient clinic appointments, adequacy of outpatient clinic equipment, timely pathologic analysis of biopsy specimens, availability of outpatient treatment scanning and operating room access [41]. In that context, Peacock et al. [17] reported a mean time from completion of tests to case presentation to the head and neck tumor board of 20.7 days (range: 1–208 days) in the US and a mean time from case presentation to the board to initiation of definitive treatment of 18.6 days (range: 1–76 days) attributing the delays in obtaining insurance authorization and in scheduling an operating room.

## 5. Implications on Prognosis and Survival

Prolonged length-time in treatment of oral cancer may impact survival as during this time-interval the tumor has the capability to increase in size and metastasize compromising the patient’s prognosis. Kowalski et al. [44] revealed the potential clinical upstaging of HNC in Brazil before initiation of treatment and demonstrated that the median 5-year survival rate of patients with clinical upstaging before treatment was significantly poorer (17.2 months) than patients without clinical upstaging and who received treatment within a short period of time (1–3 weeks) (32.7 months) [44]. Likewise, Xiao et al. [45] determined that increasing time-to-treatment initiation (TTI) in the US was significantly associated with clinical upstaging, suggesting tumor progression. Also, upstaged patients had a poorer OS compared to patients who were not upstaged (5.07 vs. 7.10 years, respectively, *p* < 0.001). When adjusting by upstaging, TTI was only a significant predictor of increased mortality beyond ≥70 days indicating that TTI itself is not a consistent predictor of mortality and is not independent of tumor progression [45]. The results suggest that survival from oral cancer depends more by the proliferative activity of the tumor (tumor progression) than by a prolonged time-interval to diagnosis [46].

Several systematic reviews have assessed the impact of time-to-treatment in HNC on OS (Table 1). The results from Graboyes et al. [47] provided support that starting postoperative radiotherapy within 6 weeks or less of surgery is associated with improved OS. Also, from the 13 included studies, nine reported a significant association between a prolonged pre-treatment interval and poorer OS, although there was a high heterogeneity among the studies regarding definitions and thresholds used for defining the diagnosis-to-treatment interval. The systematic review published by Seoane et al. [48] which included 10 studies, showed that a prolonged interval to diagnosis was associated with a moderate risk of mortality from HNC (“any delay” = RR: 1.34, 95%CI 1.12–1.61). The patient and diagnostic intervals were associated with an increased risk mortality but were not statistically significant. Only referral delay was associated with a three-fold increased mortality rate, although this pooled estimate was based on only two studies. Nonetheless, these results must be interpreted with caution as included studies were highly heterogeneous regarding tumor location, assessment and thresholds used for defining “delay” and some studies lacked to control for known confounding factors that may influenced the associations observed. Later, Seoane et al. [49] conducted a systematic review focused on patients diagnosed with OSCC reporting similar results as the previously published [44]. They also found that both prolonged patient interval and diagnostic interval were associated with a more advanced stage of disease (III or IV) at diagnosis with a higher effect size for the diagnostic interval (OR: 2.15, 95%CI 1.08–4.29) than for the patient interval (OR: 1.55, 95%CI 1.14–2.12) [49].

Recent research has focused on estimate the impact of the pre-treatment interval on OS from HNC, with findings differing across the literature (Table 2). On the one hand, Tsai et al. [50] showed that patients in Taiwan treated after 30 days from diagnosis had a lower OS rate from oral cancer when compared to those treated within 30 days; the trend observed remained the same after stratifying by initial tumor stage at diagnosis. Similarly, a prolonged pre-treatment interval (>30 days) was associated with worse OS in patients with stage III or IV oropharyngeal squamous cell carcinoma (HR: 1.12, 95%CI 1.03–1.20) and with a 2.2% (95% CI, 1.1–3.3%) increased risk of death for every 1 week increase in the diagnosis-to-treatment interval [43].

Van Harten et al. [42] found that in The Netherlands longer waiting times to initiation of treatment was significantly associated with worse OS in patients diagnosed with HNC. However, when the variable was categorized (≤30 days vs. >30 days) no significant differences in OS were observed (HR: 1.0 95%CI 0.94–1.07). Murphy et al. (US) [51] reported that a pre-treatment interval of 30 to 60 days had no impact on OS from HNC but demonstrated that the mortality risk raised substantially after 67 days and with higher time-intervals (61–90 days = HR: 1.13, 95% CI 1.08–1.19 and >90 days = HR: 1.29, 95%CI 1.21–1.38) (Table 2).

Regarding the radiation interval (time for the first to the last day of radiation), Fujiwara et al. [38] found that in the US prolonged radiotherapy duration was significantly associated with worse OS. Patients with 54 or more days of radiotherapy duration had a 5-year OS rate of 40.5%, compared to the 52.4% in patients with 49 days or less of radiotherapy duration (HR: 1.22, 95%CI 1.03–1.44). The authors discussed that may be due to the rapid repopulation of tumor clones during treatment breaks [38]. Similarly, Ho et al. [52] demonstrated no detriment to survival in the US if radiation was initiated within 40 days of surgery. Mortality risk began to increase beyond this time point, plateauing at 70 days (HR: 1.14, 95%CI 1.01–1.28) with further postoperative “delays” not worsening the prognosis. The radiation interval was also significantly associated with mortality risk from HNC which increased continuously within each day of “delay” up to 55 days (HR: 1.25, 95%CI 1.11–1.41, reference ≤40 days). The pre-treatment interval had not prognostic value on mortality from HNC after adjusted analyses.

Currently, only one study [53] has assessed the impact of the total time-interval on mortality from oral cancer. The authors found a U-shaped association, where patients with short time-intervals (24–55.5 days) and with long time-intervals (127.5–420 days) had a higher mortality than those with medium time-intervals. Higher mortality rates in patients with shorter time-intervals could be explained by confounding by severity and tumor aggressiveness [53].

## 6. Discussion

It is well known that the prognosis of patients with oral cancer largely depends on the stage of the disease at the time of diagnosis. The challenge, therefore, is to advance the diagnosis to an earlier stage, which then would result in less morbidity and a better prognosis [54]. Strategies for diagnosing oral cancer at an early stage include population screening of high-risk groups, opportunistic screening by HCP and reducing the time-intervals in diagnosis and treatment of oral cancer [55]. Since the patient interval is the major contributor to the total time-interval, priority should be given to strategies aimed at increasing public education and awareness of early signs and/or symptoms of HNC [13].

Education of the public and HCP on early cancer signs is part of the early detection approach in many countries but there have been few investigations on the impact of awareness campaigns on cancer outcomes [56]. A systematic review published in 2009, found limited evidence regarding the effectiveness of educational interventions aimed at increasing cancer knowledge and promoting early presentation at the individual and community-level [57]. Moreover, research in this field regarding HNC awareness are scarce, and the available evidence shows a modest short-term increase oral cancer knowledge at the individual level after the delivery of written information [58,59,60,61]. Short-term mass campaigns among the general population have shown inconsistent results with limited effect at increasing oral cancer awareness [62,63,64,65] and some effectiveness at increasing oral cancer screening among the high-risk population [63].

Furthermore, if educational/health interventions actually promote an early presentation and, therefore, the diagnosis at an earlier stage is still undetermined. In 2012, Scott et al. [66] reported that a pilot theory-intervention in the UK had, in the short-term, a small effect in reducing anticipated delay in seeking help for potentially malignant oral symptoms (waiting time of more than 3 months before consulting a HCP) and a recent trend analysis conducted in Northern Germany showed a slight increase in the proportion of women diagnosed with oral cancer at stage I after the implementation of an awareness campaign [67]. Although the scarcely evidence shows some positive impact on clinical and patient’s outcomes, this is still a subject that needs further research before the assessment of clinical implications.

The introduction of a fast-track policy for urgent referral for suspected cancer (NICE guidance for primary care) in the UK have shown some effectiveness in reducing the diagnostic interval from several cancers since its implementation in 2005, including HNC with a mean reduction of 21 days [68]. It is feasible that changes in policy and practice may have increased awareness of early signs and symptoms in primary care physicians leading to earlier referral to specialist or diagnostic investigation and, thus, reducing this time-interval [68]. Although many other countries have developed and implemented policies and clinical guidelines for the diagnosis and management of cancer, the impact of such strategies on the diagnosis and treatment intervals and, subsequently, on cancer outcomes have yet not been assessed and are still poorly understood.

The evidence regarding the impact of prolonged time intervals on prognosis and survival from oral cancer have not demonstrated a strong association between the patient and diagnostic intervals and poorer survival from HNC. An explanation for the heterogeneous findings could be that the relationship is rather influenced by the proliferative activity of the tumor than by delays itself, which would explain why patients who experienced a short diagnostic interval had a bad prognosis and patients who experienced a long diagnostic interval elicit good prognosis [46]. Stage of the disease at diagnosis has been identified as one of the major prognostic factors for survival from oral cancer and it seems to be dependent of the patient and diagnostic interval [49], however, the proliferative activity of the tumor is an independent factor that should be considered in future research on the topic. On the other hand, an emerging body of evidence focused on the pre-treatment and treatment intervals have shown that prolonged time-to-treatment initiation, particularly beyond 2 months, negatively impacts survival from HNC [12], which may also be confounded by the proliferative activity of the tumor as it has not been considered as an independent factor associated with prognosis and survival in previous research. Also, postoperative radiotherapy within 4-6 weeks of surgery have shown no detriment to survival [47,52] which is supported by the National Comprehensive Cancer Network Guidelines that recommend the postoperative interval to be less than 6 weeks [69].

## 7. Conclusions

The patient interval is the major contributor to the total time-interval. Unawareness of signs and/or symptoms, denial and lack of knowledge about oral cancer are the major contributors to the process of seeking medical attention, so future research should be focused at assessing the impact of educational awareness campaigns and also health interventions such as opportunistic screening or screening of high-risk groups on cancer outcomes, mainly, early presentation, diagnosis at an early stage and survival from HNC. There is an emerging body of evidence supporting the impact of prolonged pre-treatment and treatment intervals with poorer survival from HNC which needs to be further clarified by high-quality synthesis of studies. Future research on the topic must also consider the proliferative activity of the tumor when assessing the impact of time-intervals on survival from oral cancer.

## Figures and Tables

**Table 1:** Systematic reviews assessing the impact of time-to-treatment on survival from head and neck cancer.

Autor/Country/Year/Reference	Included Studies/Design	Patients	Exposure	Outcome	Results
Graboyes et al. /US/2019 [47]	18 (2007–2018) Cohort	Patients who underwent treatment of SCC of the oral cavity, pharynx or larynx	DTI S-PORT TPT	OS	**DTI** (13 studies) High heterogeneity among definitions and thresholds among studies (>20 days to >120 days) Nine studies reported a significant association between increased DTI and poorer OS. HR for DTI >30 days ranged between 1.07–1.18 and for >90 days between 1.32–1.6. The effect size of DTI on OS increased with prolonged DTI **S-PORT** (five studies) Four studies found an association between prolonged S-PORT interval (>6 weeks) and poorer OS with HR varying between 1.10–1.34 **TPT** (five studies) Four studies reported an association between prolonged TPT (≥11 weeks to ≥14 weeks) and poorer OS with HR ranging between 1.07 to 6.7. One study reported an association between increasingly prolonged TPT and a progressive decreasing trend in OS.
Seoane et al. /Spain/2016 [49]	10 (1998–2012) Retrospective Cohort	Patients with symptomatic primary oral SCC	DI	OS Disease stage (TNM)	**Meta-analyses for OS** Any delay (four studies): OR = 1.35 (0.84–2.18) Referral delay (two studies): OR = 2.48 (1.39–4.42) **Meta-analyses for TNM** Any delay (seven studies): OR = 1.66 (1.25–2.20) Patient delay (four studies): OR = 1.55 (1.14–2.12) Professional delay (three studies): OR = 2.15 (1.08–4.29)
Seoane et al. /Spain/2012 [48]	10 (2001–2010) Retrospective/ Prospective Cohort	Patients with HNC	PD PDI DD RD	OS	**Meta-analyses for OS** Any delay (10 studies): RR = 1.34 (1.12–1.61) PD (Five studies): RR = 1.67 (0.88–3.19) PDI (Five studies): RR = 1.32 (0.66–2.66) RD (two studies): RR = 3.17 (1.12–9.00) Total DD (two studies): RR = 1.04 (1.01–1.07)

**Abbreviations:** SCC = Squamous cell carcinoma; DTI = Diagnosis to treatment initiation; S-PORT = Surgery to postoperative radiotherapy initiation; TPT = Treatment package time (surgery to completion of postoperative radiotherapy); OS = Overall survival; HR = Hazard Ratio; DI = Diagnostic interval (time-interval between first symptomatic presentation and patient referral, or histological diagnosis or start to treatment); HNC = Head and neck cancer; PD = Patient delay (time from the patient’s first awareness of symptom/sign to the first consultation with a physician or dentist); PDI = Presentation-to-diagnosis interval (time from the patient’s first consultation with a physician or a dentist to the date of histological diagnosis); DD = Diagnostic delay (the sum of the patient and professional delay); RD = Referral delay (difference between the date of first symptom and the date of the referral letter transferring the patient to the secondary care level).

**Table 2:** Studies assessing the impact of pre-treatment and treatment intervals on survival from head and neck cancer.

Author/Country/Year/Reference	Study Design	Population	Exposure	Outcome	Results
Tsai et al./Taiwan/2017 [50]	Retrospective Cohort	21,263 patients diagnosed with SCC from the oral cavity from 2004–2010 identified from the TCRD	DTI	OS	>120 days (*n* = 572): HR = 1.32 (1.19–1.47) 31–120 days (*n* = 2498): HR = 1.18 (1.11–1.25) ≤30 days (*n* = 18,193): Reference
Sharma et al./US/2016 [43]	Retrospective Cohort	6,606 patients diagnosed with stage III or IV OSCC from 2003–2006 identified from the NCDB	DTI	OS	>30 days: HR = 1.12 (1.03–1.20) ≤30 days: Reference >6 weeks: HR = 1.22 (1.10–1.35) 3–6 weeks: HR = 1.15 (1.05–1.27) ≤3 weeks: Reference
Van Harten et al./Netherlands/2015 [42]	Retrospective Cohort	13,140 patients diagnosed with HNC from 2005–2011 identified from the NCR	DTI	OS	>30 days: HR = 1.00 (0.94–1.07) ≤30 days: Reference Continuously (days): the hazard of dying ascends sharply to 25 days, then the curve plateaus, until 2 months, after which increases again.
Murphy et al./US/2016 [51]	Retrospective Cohort	51,655 patients diagnosed with HNSCC from 1998–2011 identified from the NCDB	DTI	OS	≥91 days: HR = 1.23 (1.15–1.32) 61–90 days: HR = 1.08 (1.03–1.13) 31–60 days: HR = 0.99 (0.96–1.02) ≤30 days: Reference
Fujiwara et al./US/2017 [38]	Retrospective Cohort	4868 patients diagnosed with SCC of the oral cavity from 1998–2011 identified from the NCDB	DSI SRTI RTI TTP DRTI	OS	**DSI** ≥45 days: HR = 0.98 (0.88–1.09) ≤30 days: Reference **SRTI** ≥64 days: HR = 0.96 (0.81–1.15) ≤50 days: Reference **RTI** ≥54 days: HR = 1.22 (1.03–1.44) ≤49 days: Reference **TTP** ≥116 days: HR = 1.03 (0.86–1.23) ≤101 days: Reference **DRTI** ≥161 days: HR = 0.98 (0.82–1.17) ≤136 days: Reference
Ho et al./US/2018 [52]	Retrospective Cohort	15,064 patients diagnosed with HNSCC from 2004–2013 identified from the NCDB	DTI SRTI RTI	OS	**DTI** Not associated with OS (*p* = 0.387) **SRTI** ≥71 days: HR = 1.001 (0.999–1.004) 40–70 days: HR = 1.004 (1.000–1.008) <40 days: HR = 1.006 (0.994–1.017) **RTI** ≥55 days: HR = 1.000 (0.998-1.002) <55 days: HR = 1.016 (1.007-1.025)
López-Cedrún et al./Spain/2020 [53]	Retrospective Cohort	183 patients diagnosed with oral cancer from 1998–2008 from the A Coruña University Hospital	TI	OS	24–55.5 days: HR = 1.75 (*p* = 0.04) 127.5–420 days: HR = 1.55 (*p* = 0.09) 55.5–127.5 days: Reference

**Abbreviations**: SCC = Squamous Cell Carcinoma; TCRD = Taiwan Cancer Registry Database; DTI = Diagnosis-to-treatment interval; OS = Overall Survival; OSCC = Oropharyngeal Squamous Cell Carcinoma; NCDB = National Cancer Data Base; HNC = Head and Neck Cancer; NCR = Netherlands Cancer Registry; HNSCC = Head and Neck Squamous Cell Carcinoma; DSI = Diagnosis-to-surgery interval; SRTI = Surgery-to-radiotherapy interval (initiation); RTI = Radiotherapy duration interval; TTP = Total Treatment Package (surgery to radiotherapy end); DRTI = Diagnosis-to-radiotherapy end; TI = Total Interval (from first symptom awareness to the initiation of treatment).
